# Retraction: Prostate Cancer Cell Lines under Hypoxia Exhibit Greater Stem-Like Properties

**DOI:** 10.1371/journal.pone.0233203

**Published:** 2020-05-08

**Authors:** The PLOS ONE Editors

Following the publication of this article [1], concerns have been raised regarding the RT-PCR data presented in Fig 2A and the immunochemistry data presented in Fig 2B:

- Fig 2A RT-PCR, the bands in the Nanog panel 1% O2 treated PC-3 cells (lanes 1–4) appear similar to the bands in the Nanog panel 7% O2 treated DU145 cells (lanes 5–8).
- Fig 2A RT-PCR, the bands in the Nanog panel 20% O2 treated PC-3 cells (lanes 1–4) appear similar to the bands in the Nanog panel 20% O2 treated DU145 cells (lanes 5–8).
- Fig 2A RT-PCR, the bands in the GAPDH panel 1% O2 treated PC-3 cells (lanes 1–4) appear similar to the bands in the GAPDH panel 20% O2 treated DU145 cells (lanes 5–8).
- Fig 2B, the immunocytochemistry image of Nanog panel 20% O2 treated PC-3 cells appears similar to the immunocytochemistry image of Nanog panel 20% O2 treated DU145 cells.

The Commission on Research Integrity at Oslo University Hospital and Institute of Clinical Medicine, University of Oslo completed an investigation and concluded that there was evidence of scientific misconduct in the case of Fig 2A, and negligence in the preparation of Fig 2B.

In light of the above concerns and in line with the institution’s recommendation, the *PLOS ONE* Editors retract this article.

YM, DL, JL, GK, and JMN did not respond. KA and TS agreed with the retraction. ZS did not agree with the retraction.
